# Surgical Immunization Compared with Susceptibility and Predisposition to Infection*Read before the Medical Society of Virginia, September 10, 1896, at Rockbridge Alum Springs.

**Published:** 1896-10

**Authors:** J. McFadden Gaston

**Affiliations:** Atlanta, Ga.; Professor of Principles and Practice of Surgery, Southern Medical College; Ex-Chairman Section on Surgery and Anatomy, American Medical Association


Southern Medical Record.

A MONTHLY JOURNAL OF MEDICINE AND SURGERY.

VOL. XXVI. ATLANTA, GA., OCTOBER, 1896. No. 10.

### Original Articles.

### SURGICAL IMMUNIZATION COMPARED WITH SUSCEPTIBILITY AND PREDISPOSITION TO INFECTION.*

BY J. McFADDEN GASTON, M. D., ATLANTA, GA.

Professor of Principles and Practice of Surgery, Southern Medical College; Ex-Chairman Section on Surgery and Anatomy, American Medical Association.

The term surgical immunization refers to that change in a part or the whole of the organism, by which there is conferred an exemption, to a greater or less degree, from the consequence of any surgical procedure. This may come about without any therapeutic agency whatever, and may be the result of ordinary or normal development of the organization. On the other hand changes may ensue from pathological processes of various kinds which shall have a protective influence against any untoward influence from surgical interference; and inflammation may prove a safeguard subsequently against injury.

In the paper which it was my privilege to read before this Society last year, I dwelt upon the practical features of pyemia and septicæmia; and the consideration of immunity and susceptibility on this occasion may serve as an explanation of some obscure points in my former contribution.

It must be apparent that the most essential conditions for comprehending surgical pathology are embodied in the recip-

* Read before the Medical Society of Virginia, September 10, 1896, at Rockbridge Alum Springs.

rocal relations of immunity and susceptibility, so that I can not present to your Society a subject of more importance to the surgeon and general practitioner than that selected for our consideration at present. The members of this Medical Society are requested to favor me with their attention and to analyze the data, so as to discuss the various points of principle and practice which are involved in my paper. The outcome of a thorough discussion of these matters must promote the adoption of conservative measures in surgery, and I am pleased to repeat the words of the editor of the *Virginia Semi-Monthly*, that, "headstrong empiricism in surgery is the thing which is fast bringing that great science and art into discredit."

Elaborate papers upon immunity and susceptibility, with special reference to surgical cases, were presented by Dr. Roswell Park and Dr. Joseph Ransohoff at the meeting of the American Surgical Association, in May 1896, at Detroit.

The conclusions of surgical importance reached by the former, imply that, "the surgeon in emergency cases has to do the best he can, not merely with the means at hand, but with the tissues at hand. When time may be afforded, it is the surgeon's bounden duty to so order the habits, the diet, the urroundings, and the preparation of his patient, as to restore is tissues and vital fluids, so far as possible to their normal condition, before he interferes with their functions by an operation." Since the inauguration of the so-called antiseptic era, Dr. Park contends that in our enthusiasm for combatting infection from without, we have lost sight of, and, to a great extent neglected, a most important truth, which we can not afford to disregard, by not preventing infection from within. In this connection Dr. Ransohoff states that the extent to which we are in position to influence this entero-sepsis affects the surgeon less than it does the general practitioner. It is exceedingly doubtful, whether by any safe medication known, the intestinal canal can be made aseptic. Nevertheless, he says, "no major operation should be undertaken, if time permits, without careful attention to this most important of the emunctories."

He claims that the position of a trauma or operation very signally influences the susceptibility to surgical infection. It is alleged by him that parts freely supplied with arterial blood and from which, by many channels, the return circulation is insured, do not easily become the sites of the hypostatic hyperæmias, which are often the immediate precursors of surgical infections.

A very important point of view, in consideration of the question of susceptibility and immunity to surgical infection, is that of operative infection. With a proper comprehension of the dangers of such infection, they may be largely avoided and Ransohoff's motto is *non nocere*.

In my discussion of the papers I noticed some instances of immunity to traumatism conferred by previous inflammatory processes, and drew attention to the results of increased susceptibility to certain forms of inflammatory action from slight superficial injuries. The development of lymphangitis from a slight abrasion, involving the minute ramifications of the nerves and capillaries, was attributed to the ganglionic element which is supplied to the rudimentary structure of the connective tissue.

The peculiar consequences of punctured wounds and small lacerations of the integument, developed in the form of tetanus, are dependent upon ptomaines, and it is very rare to find suppurating wounds followed by tetanus.

There must exist not simply a susceptibility on the part of the general system, but a very marked predisposition to take on an impression from a local injury, where lymphangitis or tetanus is developed in such lesions.

Under different conditions, it is found that pyemia and septicæmia follow traumatism.

A distinction should be recognized between the normal and physiological condition of the nerves and blood-vessels in recent injuries, as compared with the abnormal and pathological state, resulting from inflammation or other structural disorders of any portion of the organism. A proper discrimination between these results of violence and disease forms the basis of treatment for the different stages of wounds. A primary dressing is made with a view to the prevention of trouble beyond that of traumatism; the secondary measures may require a consideration of the changes in the tissues, and the consequent general derangement of the organism, including both the vascular and absorbent systems. All changes in the organism of a secondary character depend on an impression upon the nerve centers from the local injury, which is propagated by the efferent nerves and the ganglionic nerves to the entire organization, constituting what is styled constitutional disturbance. The action and reaction observed between traumatic lesions and constitutional derangement develop a general effect upon the secretions.

To appreciate the changes wrought upon the tissues by inflammatory processes, it becomes necessary to look into the composition of the different textures of the organism. The cellular constituents of muscles, blood-vessels, nerves, serous and mucous membranes, undergo modifications from inflammation, which render these structures less disposed to resent the impression made by a surgical operation, than is observed in normal tissues. How far the presence of microbes and the process of phagocytosis may operate in modifying the condition of the tissues, under the course of inflammation, must be considered in determining upon immunization.

While there is sufficient ground to account for the development of inflammation in some cases without tracing it to bacterial origin, and perhaps in other instances the bacteria may be a concomitant rather than a cause of inflammation, yet the experimental results go to prove that in some manner not very well defined changes take place, by which ptomaines are produced in connection with leucomaines.

As a counterpart to the agencies of immunization it devolves upon us to study the modification of susceptibility and predisposition to infection. I employ the word infection, in its ordinary signification, as the invasion of a wound by septic germs, such as interfere with the normal progress of healing or induce suppuration. In Foster's Encyclopædic Medical Dictionary it is defined as "the act or process by which disease s set up in an organism, by the implantation of morbific germs from without; or of a part of the organism, by the conveyance of such germs from another part." The advanced bacterial school may consider this term, "germs," as entirely too general in this day of exact designation of diseases by their appropriate bacilli, but for the purpose of understanding the relations of these different modifications of the organism, it is not requisite to go into minute details.

I make the distinction between susceptibility and predisposition as passive and active conditions, demanding, respectively, greater or less force to produce an effect. The observance of hygienic precautions in all respects affords the best protection against diseases, and in like manner the avoidance of all vicious habits which may impair the powers of the organism, places the body in the most favorable condition to resist the depressing influence of surgical operations.

It is becoming recognized generally by the profession that immunization from tuberculosis results largely from the environment of the individual, and that in its incipient stage restoration of vigor by open air life and exercise, accomplishes a cure in some cases. Quite a change has come over the surgical practice in local tubercular developments, and while the progress of the disease in a particular part may be arrested by operative measures, the tubercular diathesis must be corrected by general treatment. The predisposition to tuberculosis can not be relieved by a surgical operation upon the diseased structures, but must be corrected by remedial agencies acting through the absorbent and secretory organs.

The general belief that experimentation upon inferior animals in their normal condition of health may be relied on as a guide in performing surgical operations upon the human being, does not take into the account an important factor connected with the pathological condition of the structures. There may exist such modifications from acute inflammatory processes as to preclude an operation, or there may be present results of chronic inflammation of the tissues which shall prove advantageous by securing tolerance of surgical interference. The robust, athletic subject, with strong proclivities to hyperæmia, meets with an accident while enjoying perfect health which interferes materially with the vital functions, and the local injuries call for operative measures at once. It is evident that a patient under such circumstances will not bear a surgical procedure so well as one who has become accustomed to the wear and tear of a slow inflammatory process which prepares the tissues for a resort to the knife.

Ashhurst states that "if the intermediate or inflammatory stage has come on, operative interference must, if possible, be postponed until the inflammation has measurably subsided, and until the patient's condition has become assimilated to that of a case of chronic disease rather than of traumatic lesion."

It is true, however, that secondary amputations give a somewhat greater fatality than primary amputations, being 45.5 per cent. of mortality in the latter, with 32.3 per cent. of mortality in the former, out of 2755 cases tabulated from twenty-one different reports prior to the days of antiseptic surgery.

The general results given in the supplement to the International Encyclopædia of Surgery from amputations at all stages, in the practice of Ashhurst in 253 cases, is 26.4 per cent. of mortality.

The statistics of cases in which amputation has been done for diseases, show a mortality of 26.8 per cent., while those for injury reach 39.8 per cent. of mortality.

There is a peculiar feature of nervous depression known familiarly as shock, which results from surgical operations, not otherwise of a serious nature. In undertaking any operative measure it is prudent to forestall a tendency to this form of vital prostration by imparting tone to the nerve centers and various medicaments have been resorted to, before entering upon operations, with a view to prevent shock, while similar agencies have been used to restore patients from the threatened collapse attending cases of profound shock.

As prophylaxis is far preferable to therapeusis, it of course is very desirable to anticipate the trouble and prevent it if possible, and the most satisfactory proceeding is to keep the patient for some hours or even days, when practicable, under the sustaining influence of strychnine by hypodermatic injections of the $\frac{1}{30}$ gr. at such intervals as may be convenient. Within an hour of the time for an operation, a whiskey or brandy toddy may be given, and in half an hour, inject sul. morphia ¼ gr., and sul. atropia $\frac{1}{150}$, followed by A. C. E. anesthetic.

It is very desirable that the profession shall discover some means of averting the fatal consequences which occasionally follow the administration of anesthetics.

Until the precise lethal operations of the inhalation of sulphuric ether and chloroform is understood we can not proceed philosophically towards providing a remedy. But I am favorably impressed with the use of oxygen in view of the change which the arterial blood undergoes in the collapse of anesthesia corresponding to that of asphyxia. The dark color of the arterial blood under anesthesia struck me forcibly upon first using it, shortly after it was introduced by Simpson, and yet there has been little notice taken of the absence of oxygenation of the blood under anesthesia. It seems rational to inhale oxygen or force it into the lungs when a patient becomes comatose from asphyxia, but the effects of anesthesia collapse are so sudden as to afford little opportunity to apply the remedy, even when it is already at hand.

The supposition that immunity resulted from local applications to the field of operation led to the use of antiseptic dressings with a view to prevent contamination. But further observation convinced operators that no favorable influence was exerted by germicidal washes upon normal structures, and that the only indication for antiseptic applications is to combat existing septic conditions.

A very interesting illustration of the effect of the development of modern surgery is given by Henggeler in 276 cases of strangulated hernia at Zurich from 1881 to 1894. During the period of the use of carbolic acid as an antiseptic—*i. e.*, from 1881 to 1885, 38.15 per cent. of the cases died; during that of bichloride, *i. e.*, from 1885 to 1893, 21.1 per cent. died, and since the introduction of aseptic methods only 16.3 per cent have died.*

In 48.4 per cent. of the 64 fatal cases, the cause of death was gangrene of the intestines and peritonitis, but there is nothing said as to the influence of antiseptics in producing these troubles. It is the accepted doctrine now that asepsis secures greater immunity from contamination than antisepsis in surgical operations upon structures in their healthy physiological state, and few operators follow the practice of five years ago by resorting to germicidal applications to fresh cut surfaces.

I occupy the same position which has been held by me during all the vicissitudes of the war against germs, and the most advanced views of bacteriologists are exactly in unison

*Annals of Surgery, July, 1896.

with the doctrine I have advocated from the outset of this controversy. It has not been a tacit acquiescence in what was maintained by others, in regard to the hurtfulness of all germicidal applications upon fresh cut surfaces, but at all times and in opposition to the tenets of colleagues with whom my lot was cast, I have openly spoken out my convictions as to the injurious effects of carbolic acid and the bichloride of mercury solutions in connection with operations on normal structures, and that they were only admissible in septic conditions.

There is a phase of septic development to which potent germicides are applicable, and when a local source of contamination exists, whether it is associated with bacteria, fungi or ptomaines, the most vigorous use of antiseptic measures is demanded. It is evident that septic processes, which uncontrolled would lead to septicæmia, are arrested in many instances by correctives of various kinds, and a prompt resort to antiseptics, and germicides is indicated to arrest the progressive contamination of the general organization from the local septic process.

A normal, healthy structure does not call for the application of germicides, but when the tissues have undergone contamination, antiseptic applications are requisite, and it is held by some strenuous opponents of poisonous drugs that innocuous agents may be employed as antiseptics with a satisfactory result. Yet it is the practice still with some members of the profession to employ solutions of carbolic acid and corrosive sublimate upon delicate tissues without regard to the serious results of their absorption.

In my address at the opening of the Southern Medical College in October, 1888, upon aseptic, antiseptic and septic surgery, all the practical appliances of a surgical order were embraced under these divisions. The first embodies all those means which come under the heading of prophylaxis, and looks to the avoidance of hurtful agencies, so as to be assured that if a measure does no good it shall do no harm. It is the highest type of conservatism to preserve the organs or tissues involved in an operation against any kind of mutilation or injurious impression from other sources.

Whatever may cause impairment or disability of the constituent elements of a part, whether in the influence of a disease upon the tissues, or from the effects of a surgical operation with the accompanying local applications, should be discounted from the result. The true aim and end of surgery being to preserve and not destroy a part which may be diseased or injured, all the processes of asepsis that look to its restoration are of the greatest importance to the surgeon, and commend themselves to his adoption under all circumstances.

Antiseptic applications in surgery differ materially in their aim and results from aseptic measures. The hypothesis of the bacterial origin of inflammatory processes, with suppuration and degeneration of structures after injuries, has led the medical philosopher and scientist to search for the separate germs of the various disintegrating processes, and to point out the special microbe of distinct diseases. That we find micrococci, spirilli, spores, fungi and ptomaines associated with certain disorders of the animal economy, indicates that they have a share in their development, but whether the evidence of their presence warrants a resort in advance to germicides, has now received an adverse decision by bacteriologists.

The sphere of antisepsis, as now recognized, is to correct a tendency to or a development of degeneration and disintegration of living structures, resulting from contamination in any form. If we are able to discover the causative element and neutralize it by antiseptic measures of a topical or a constitutional nature, this is eminently proper.

But in default of identification of the cause, it devolves upon us to combat the injurious effects of the vicious impression already made upon the organism, and this is the special province of antiseptic surgery, instead of seeking to anticipate trouble by the use of germicides.

The claim, as put forth formerly for antiseptic surgery, included many measures supposed to be preventive, which proved to be disturbing influences, so that instead of protecting the organization, by immunization, they produced derangement of the system and led to septic developments. Having the courage of my convictions based upon investigation of this subject, I have protested against a blind acquiescence in what has been erroneouly styled antiseptic surgery, and it gives me great satisfaction to know that my position is sustained by the best men in the profession at the present time.

The cases of poisoning resulting from even the most cautious use of carbolic acid and bichloride solutions call for their abandonment in surgery. It is this class of agents which I have placed under the division of septic surgery, instead of antiseptic surgery.

The point is settled beyond controversy that they are potent germicides, but in the race after micro-organism, more damage was done to the structures in which they are supposed to be located, than the microbe is capable of doing if let alone. The great dangers from the contact of solutions of carbolic acid and corrosive sublimate with recently cut surfaces are now so well known to the profession throughout the world, that it is unnecessary to specify instances to any reader of current literature. We feel, therefore, that surgeons who continue to employ solutions of these poisonous drugs are without a shadow of excuse, and should realize that they are not practicing true antiseptic surgery, but septic surgery. The consideration should come home to them that asepsis has taken precedence in surgery involving normal structures.

There has cropped out recently in a meeting of railway surgeons some views indicating that there is something calling for antiseptic surgery in the treatment of railway accidents which does not exist in the case of other injuries. Why lacerated and contused wounds received in a collision or in any other manner connected with a railway should differ from a similar injury inflicted under other conditions is quite incomprehensible. The great point made that the difficulties in the way of practicing asepsis renders it important to adopt antiseptic measures, preliminary to carrying out aseptic treatment, does not avail against the claim of bacteriologists of good standing, demonstrating that the use of germicides in uncontaminated structures proves generally injurious to the tissues.

It looks like that sophistry which would do evil that good may come, and I am at a loss to understand a plea for this line of treatment in railway surgery.

There has been such a variety of injuries from accidents in riding bicycles, that in this class of wounds, as in those connected with railway service, there are supposed to be some special features by which they are to be contra-distinguished from other lesions of the tissues. A writer in the *British Medical Journal* of July 25, 1896, states that "the cycle graze is very troublesome to heal. The two upper layers of the skin over some bony prominence are killed by the blow from the fall, and the resulting sore, if neglected, often remains open for weeks." Other comments are made upon the distinctive character of the wound, but the author admits that the injuries sustained from cycle accidents do not differ in any essential particular from those caused by other falls from swiftly moving carriages. We may anticipate that the day, is not far distant when we shall have cycle doctors and baseball doctors set apart for special work in each of these departments.

It is prudent policy to rely upon the *vis medicatrix naturæ* to a large extent in the management of violent traumatic accidents, and there is a masterly inactivity which avails much towards the restoration of structures that have been seriously injured, without material impairment of vitality.

The province of asepsis in such cases is proper adjustment of the structures and the prevention of everything likely to interrupt the curative process. It is doubtful if anything is better than the blood plaster from the saturation of a roller around a fresh cut on either the hand or foot. The parts are in their normal state, with a good prospect of healing if let alone, so that meddlesome surgery can only interfere with the curative process, and "hands off" is the true policy in this class of cases. With the lights before us and the resources at our command, the surgeon is warranted in undertaking to save limbs, which under the old regime were necessarily sacrificed. But we should not try to save a dead member; it being our duty to lop off what can not be restored to life.

Asepsis is the prevention of all' contamination from within or without, in the treatment of wounds, and the most extensive traumatism may yield a good result with the proper use of aseptic measures. Among aseptic resources the most important then is cleanliness, which John Wesley declares to be next to godliness. The idea conveyed by freedom from dirt or filth applies not alone to the absence of any foul or extraneous matters from the wound, but its complete removal from the surroundings of the apartment. Of course, the clothing and bedding of the patient should be free from blot or stain or any such thing.

Yet, withal, it has been questioned whether those who have been accustomed to live in the midst of filth can be entirely removed from such surroundings without a protest of nature against such a revolutionary measure. It is even held by some gynecologists that women whose houses are not models of hygienic requirements bear up under a laparotomy at their own quarters better than in the best regulated hospital.

My personal experience in undertaking the management of cases requiring extensive incisions for the removal of large ovarian tumors in private quarters has been entirely satisfactory, and I doubt the advantages claimed by some operators for hospital surroundings in laparotomy. One of the gynecological staff at the Grady Hospital remarked in my presence, on one occasion, that his results in abdominal sur[illegible]ry of females had been more favorable in those cases operate[illegible] upon at their homes, however humble, than in the hospital. I have not at hand the statistics of the gynecological work in the Grady Hospital, but the general impression of the profession in Atlanta is that there is a much larger mortality in laparotomies done there than in the same kind of operations in private practice. The most feasible explanation of the increased mortality in hospital practice is the comparative immunity conferred by observing, more or less, the habits of life to which the patient has become accustomed.

I witnessed a striking illustration of the effect of habit, a few years since, in a lady of sixty years of age, upon whom I operated for the removal of an immense cystic tumor of the ovary. Everything progressed well for a day or two after the laparotomy when the patient's listless and depressed condition attracted the attention of all around her, and upon inquiry, at last it cropped out that she had given up her habit of using snuff since the operation, of her own accord. Dr. G. G. Roy, being the family physician, was assisting in the case and after treatment of the patient, and directed her at once to resume the use of her snuff. Within twenty-four hours afterwards, our patient had undergone a most favorable change, and continued to improve without any further drawback until completely restored to health.

There is no doubt, also, in regard to the use of spirituous liquors habitually by those who undergo surgical operations, and a sudden interruption of this indulgence lays the patient liable to disturbances of various kinds.

On the contrary, it is well understood that those who have been excessive drinkers of ardent spirits have their vitality so much impaired by its effects upon the organism that they do not resist the shock to the general system from operation so well as others who have led lives of abstinence. The immunity of habit avails for those who have indulged moderately in the use of intoxicants, and a modified use after surgical operations is indicated. But this even gives no security corresponding to the immunization of a vigorous normal state of the organization, such as is found in perfect health and proper performance of all the functions of the body.

The law of habit comes into play still more distinctly in those who are victims of the use of opium to an extent that their nerve-centers are very materially affected by it. In all such it may be laid down as a rule for the guidance of the surgeon, that the habit must be respected, by its use for a time after operations are performed.

As a corollary to this presentation of the claims of immunization as related to susceptibility to infection, the following inferences may be drawn:

1. That various agencies are at work in rendering the human organism to a greater or less extent free from the injurious impressions of surgical procedure.

2. That local and constitutional influences operate in conferring immunity, and the environments of individuals, with their habits and customs of life, exert great control over the vital powers.

3. Certain marked changes in the conditions of the nervous system, constituting shock, in course of surgical operations, may be averted by proper measures in advance, and in default of such precautions, should be corrected by vigorous means of treatment.

4. That the immunity for normal structures in operative work, which was supposed to be given by germicidal solutions has proved to be a delusion and a snare, and that they are only admissible in septic contamination of the tissues.

5. That a preliminary examination of all the functions of vital organs should precede surgical operations of every kind, and that efficient correctives should be resorted to for their derangements. The issue of the case depends materially upon proper means of preparation for an operation.

6. It is not essential for the management of a surgical case that the patient be placed in a hospital, but cleanliness in private quarters, with proper nursing, may secure entirely satisfactory results, by conforming to the ordinary surroundings of the patient.

7. A thorough comprehension of the reciprocal relations of immunity and susceptibility should lead to the adoption of conservative measures in the practice of general surgery, and the use of the most radical and aggressive measures when indicated by the nature of the case.

8. Those appliances which may promote surgical immunization should be adopted, and those means which lessen susceptibility and predisposition to infection are warranted in all cases of surgical interference.

